# Eikonal Phase Retrieval: unleashing the potential of fourth-generation sources for enhanced propagation-based tomography on biological samples

**DOI:** 10.1107/S1600577525005223

**Published:** 2025-07-17

**Authors:** Alessandro Mirone, Joseph Brunet, Theresa Urban, Hector Dejea, Leandre Admans, Renaud Boistel, Morgane Sowinski, Pierre Paleo, Henry Payno, Stijn E. Verleden, Camille Berruyer, Elodie Boller, Claire L. Walsh, Peter D. Lee, Paul Tafforeau

**Affiliations:** a European Synchrotron Radiation Facility 71 Avenue des Martyrs F-38000Grenoble France; b Department of Mechanical Engineering University College London London United Kingdom; c UMR 7179, Muséum National d’ Histoire Naturelle, 57 rue Cuvier, CP 55, 75005Paris, France; d Laboratoire d’Acoustique de l’Université du Mans (LAUM), UMR 6613, Institut d’Acoustique-Graduate School (IA-GS), CNRS, Le Mans Université, Avenue Olivier Messiaen, 72085Le Mans, France; e Antwerp Surgical Training Anatomy and Research Centre University of Antwerp Wilrijk Belgium; f Department of Thoracic and Vascular Surgery and Pneumology, University Hospital Antwerp, Edegem, Belgium; Brazilian Synchrotron Light Laboratory, Brazil

**Keywords:** phase-contrast tomography, eikonal approximation, synchrotron imaging, iterative phase retrieval, propagation-based imaging

## Abstract

The evolution of synchrotrons towards higher brilliance beams has increased the possible sample-to-detector propagation distances for which the source confusion circle does not lead to geometrical blurring. This makes it possible to push near-field propagation-driven phase-contrast enhancement to the limit, revealing low-contrast features that would otherwise remain hidden under excessive noise. Until now, this possibility was hindered in many objects of scientific interest by the simultaneous presence of strong phase gradient regions and low contrast features. The strong gradients, when enhanced with the now possible long propagation distances, induce such strong phase effects that the linearization assumptions of current state-of-the-art single-distance phase retrieval filters are broken, and the resulting image quality is jeopardized. Here, we introduce a new iterative phase retrieval algorithm and compare it with the Paganin phase retrieval algorithm, in both the monochromatic and polychromatic cases. In the polychromatic case the comparison was done with an extrapolated Paganin algorithm obtained by reintroducing, into our phase retrieval algorithm, the linearization approximations underlying the Paganin forward model. Our work provides an innovative algorithm that efficiently performs the phase retrieval task over the entire near-field range, producing images of superior quality for mixed attenuation objects. Our tests on data with shorter propagation distances show that the artifacts, which our algorithm effectively addresses, are present already in more standard third-generation synchrotron setups. This highlights the potential broad applicability of the Eikonal Phase Retrieval method.

## Abstract 2

A new iterative phase retrieval algorithm, Eikonal Phase Retrieval, tackles large phase gradients and yields higher-quality propagation-based X-ray tomography of strongly heterogeneous biological samples.

## 1. Introduction

Among the phase-contrast techniques employed at synchrotrons, propagation phase-contrast micro-tomography (PPC-µCT) is the most commonly used, both in terms of devoted beamlines and imaging throughput. This is due to its simplicity, and to its photon cost efficiency, with a PPC-µCT implementation consisting of a simple detector downstream-translation applied to a standard radiography setup. While the initial pioneering phase employed monochromatic beams (Snigirev *et al.*, 1995 ▸; Antoine *et al.*, 2002 ▸), it has been found that using a broad bandwidth beam generated by a wiggler or a bending magnet, with only filters and no crystal or multilayer monochromator, is suitable for propagation phase-contrast techniques (Wilkins *et al.*, 1996 ▸; Cianciosi *et al.*, 2021 ▸). In contrast, far-field techniques require more sharply monochromated beams with longer temporal (longitudinal) coherence. This, however, comes at the cost of reduced photon flux and typically requires either several distances of propagation or displacement of masks in the beam. In these techniques, speckles and fringes result from the interference of radiation originating from distant parts of the wavefront, and, consequently, different parts of the sample, following paths of varying lengths (da Silva *et al.*, 2017 ▸).

In PPC-µCT, most of the interference fringes disappear, smeared out by the short temporal coherence, but the effect of the transversal derivatives of the wavefront phase remains. These phase derivatives are induced by the phase density gradients of the sample. Since the derivative is a local operator acting at a given point of the wavefront, the transversal phase derivatives effects remain with short longitudinal coherence (Pogany *et al.*, 1997 ▸). A familiar example in the visible domain would be the heat shimmers seen above a sun-bathed hot asphalt road. Such shimmers result from propagation-enhanced phase contrast induced by the vertical density gradient of the air. They become visible when the effects of the small deviations of the light are amplified by a long propagation. In the X-ray domain the ratio between the imaginary part and the decrement of the refraction index is positive (Thompson, 2001 ▸). Consequently, the focusing (defocusing) effect of the phase second derivatives, the lensing effect, intensifies the brightness (darkness) of a transparent (absorbing) feature. The visibility of small phase gradients increases with propagation distance as long as their induced angular deviation is larger than both source and detector pixel angular diameters as seen from the sample. Thanks to this increased visibility, propagation phase-contrast imaging enhances the signal above Poisson noise without increasing the absorbed dose, which is often detrimental to the samples. Moreover, it also increases the signal ratio over systematic errors, such as those due to irregularities in the detection devices, or inhomogeneities in the beam profile. Simply extending the counting time in absorption-based imaging would not suffice to reduce these systematic effects. The formalism that is applied in the analysis of propagation phase-contrast radiographs aims at deducing the intensity distribution of a fictive wavefront plane situated immediately after the sample, given the detector measured intensity. The latter contains strong phase effects, with the propagation-enhanced signal strengthened over the statistical noise and systematic errors, while the retrieved intensity, deduced from such phase-enhanced signal, is simply what one could measure based on the sample absorption effect only, and has reduced noise. Once the intensity at the fictive wavefront plane is known, the reconstruction problem is reduced to one of standard absorption tomography, but with an improved signal over noise ratio compared with what one would measure experimentally without the long propagation distance. The simplicity of the experimental setup, combined with the drastic improvement of the signal-to-noise ratio, explains the wide usage of propagation phase-contrast techniques (Zhou & Brahme, 2008 ▸).

One key application area of tomography at synchrotron sources is the imaging of biological samples, with about one-third of all the accepted proposals at the three PPC-µCT ESRF beamlines BM18, ID19 and BM05, over the last year, for a total of 2032 h of beam time (ESRF Users Office, 2023 ▸). One of the most fascinating properties of biological samples is that their structural hierarchy is ubiquitous at all scales. This implies that the signal contains both weak and strong components. For instance, a sample containing tiny blood vessels, visible thanks to the weak refractive index differences between tissues and liquids, might also contain sharp edges at the interfaces of bone structures. Increasing the propagation distance may enhance visibility of the weak components, but likewise it also increases the signal from the strong components.

We demonstrate that this enhancement may exceed the validity limits of current algorithms, generating streak artifacts in the reconstruction. In this work, we extend the theory underlying PPC-µCT algorithms to improve reconstruction quality in the case of strong phase gradients and large dynamics in transmitted intensities. The algorithm is based on the eikonal approximation. The term *eikonal* first appeared in the physics of wave phenomena [waves in channels, optics, quantum mechanics (Landau & Lifshitz, 1977 ▸)], describing wave amplitude integrals along minimum-action paths. We adopt a similar approach here, naming it Eikonal Phase Retrieval (EPR). Moreover, as we explore the high-contrast regime, we also introduce an analytical ansatz for the scintillator point spread function, which we use in the data preprocessing step to deconvolve the scintillator’s diffused-light background. This reduces low-frequency artifacts from non-uniform X-ray illumination on the scintillator. We call this Scintillator Light Deconvolution (SLD).

## 2. Results

The EPR and SLD methods were validated using four biological samples: a sheep head (purchased from a butcher and subsequently skinned), a giant frog of the species *Leptodactylus pentadactylus* (smoky jungle frog, collection number IRSNB 391F), an adult human right lung [University of Antwerp ethics committee (approval: EDGE 001693)] and a rabbit bone. The sheep head and the frog were preserved in 70% ethanol; the lung was stored in 4% formalin. All samples, with the exception of the rabbit bone, which was scanned in air, were prepared following the protocol for hierarchical phase-contrast tomography (HiP-CT) (Brunet *et al.*, 2022 ▸) and subsequently scanned using the HiP-CT technique (Walsh *et al.*, 2021 ▸).

The sheep head posed significant challenges due to its strong absorption contrast, caused by the extended path lengths through dense bone and the pronounced phase gradients at both the external surfaces and internal structures. In contrast, the frog exhibited lower absorption and finer structural features. The lung, when reconstructed using standard phase retrieval methods, presented problematic streak artifacts caused by the presence of bubbles within its interior, whereas, for the rabbit bone, the artifacts originated from the bone’s inner cavities. These samples are representative of the research conducted at beamline BM18, particularly within the framework of the Human Organ Atlas Project (Lee *et al.*, 2021 ▸).

We performed tomography of the sheep head at an average BM18 beam energy of 109 keV using a pixel size of 28 µm and a propagation distance of 30 m.

**Figure 1:**
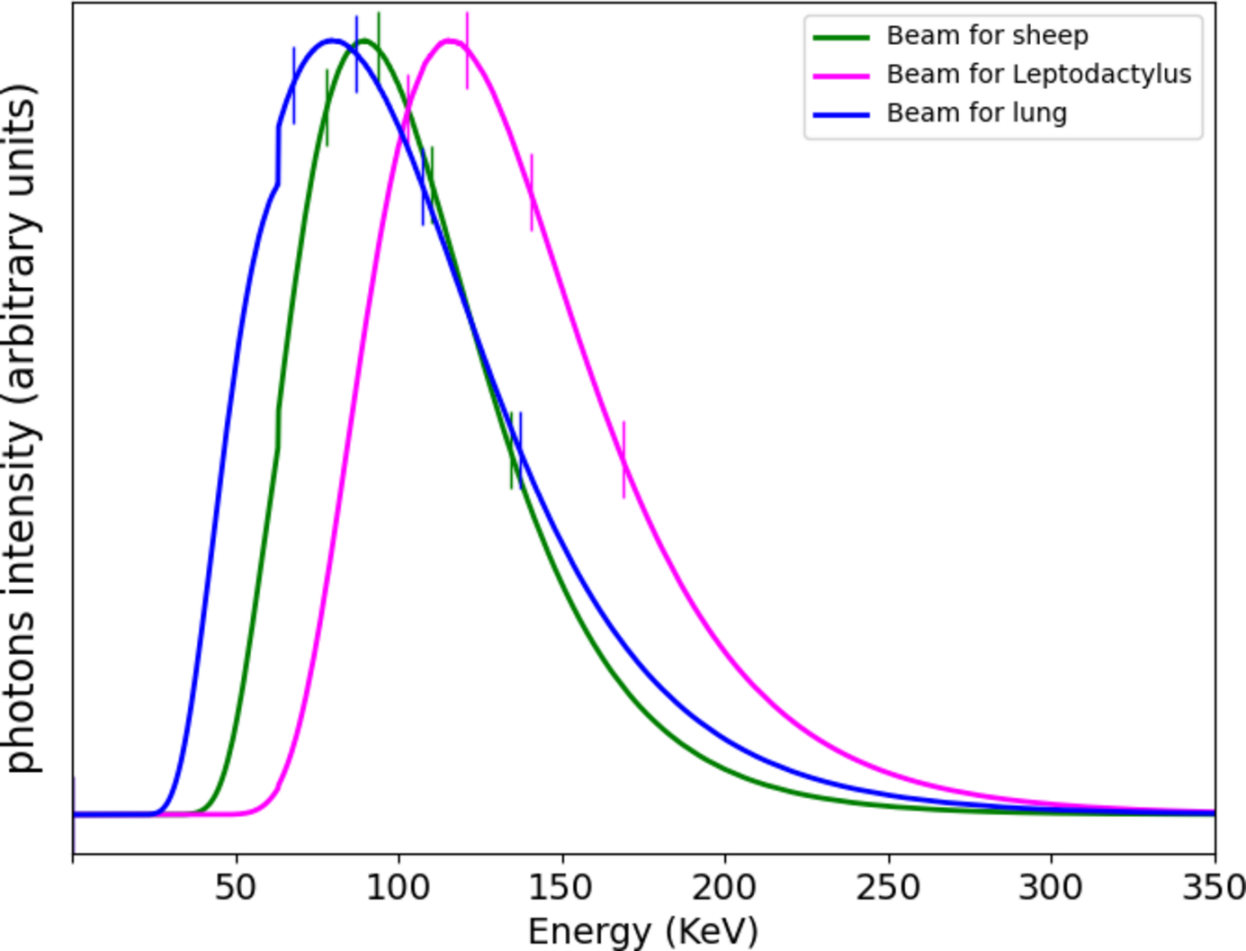
Spectral shape used for the different samples. Blue curve: adult human lung experiment. Green curve: sheep head experiment. Magenta curve: *Leptodactylus pentadactylus* experiment. The vertical lines delimit the intervals used in the discretization of the spectral shape. The spectra used for the rabbit bone is close to the one used for the sheep’s head and has been omitted in the plot.

Fig. 1 ▸ shows the relevant X-ray spectra. In the HiP-CT method the radiographs are taken of a sample immersed in a cylindrical jar, larger than the field of view, filled with an embedding fluid (ethanol for the frog and the sheep, 4% formalin for the lung), and the sample signal is obtained dividing the measured intensities by the radiographs of the same jar filled with the fluid only. The figure shows the beam effective spectral shape after conversion by the scintillator and detector signal collection, taking into account the absorption paths and conversion and detection efficiency. The spectra reaching the sample depends on the wiggler parameters and the filters which are used to attenuate the beam and change its spectral shape (Tanaka & Kitamura, 2001 ▸; Sánchez del Río & Dejus, 2011 ▸).

**Figure 2:**
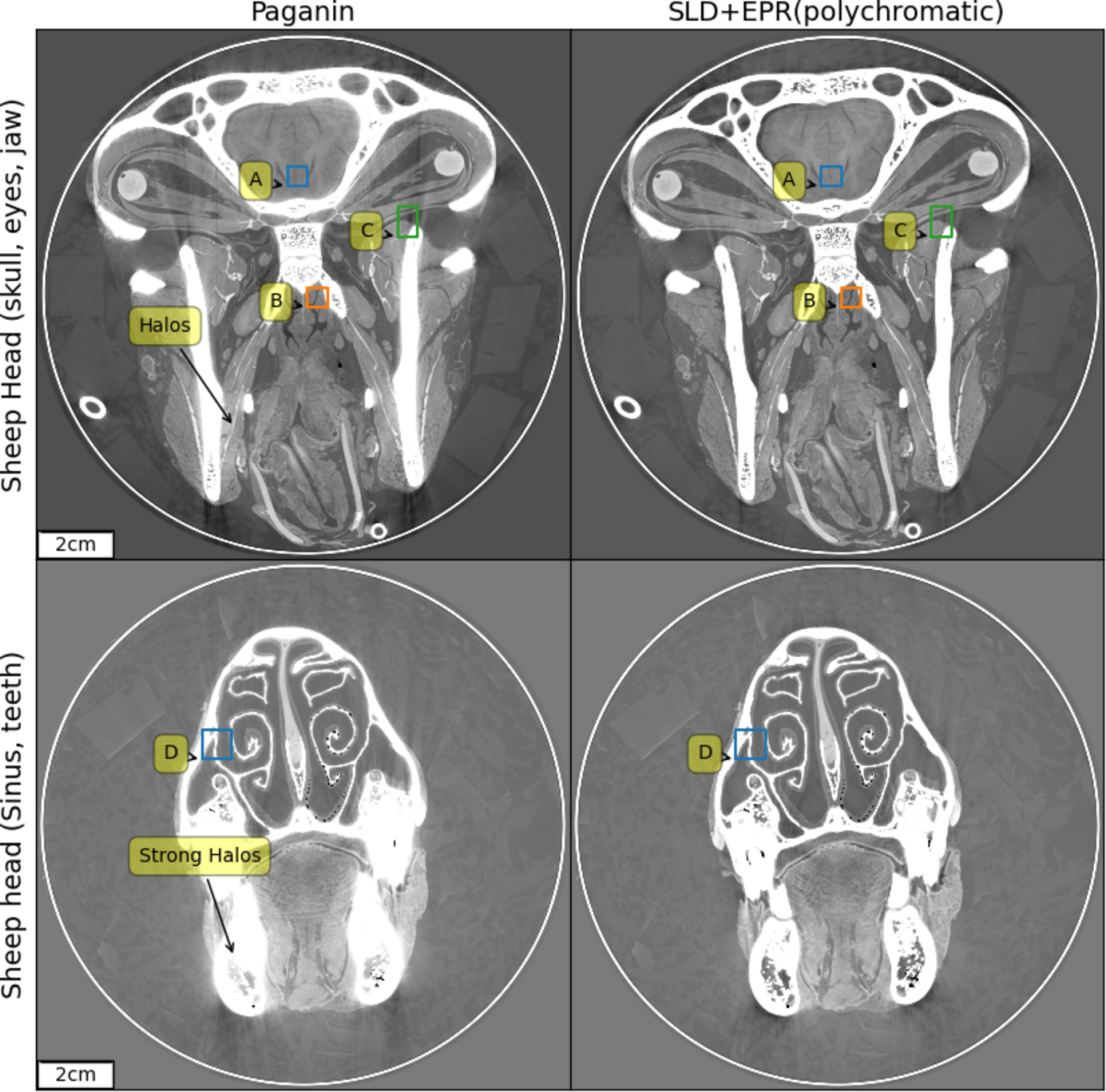
Sheep head HiP-CT scan at an average detected energy of 109 K eV, 5 K × 5 K pixels images. Left, the Paganin *et al.* algorithm at the average detected beam energy. Right, the result obtained using the algorithms introduced by the present work. For EPR we have considered five spectral points across the BM18 detected spectrum and we have applied SLD for a tail containing 30% of the total signal and a damping length of 80 pixels. We used the refractive indexes for 75% hydroxyapatite, 25% collagen. The regions marked A, B, C and D are shown in detail in Fig. 3 ▸, highlighting a detailed comparison between EPR and Paganin *et al.* methods.

**Figure 3:**
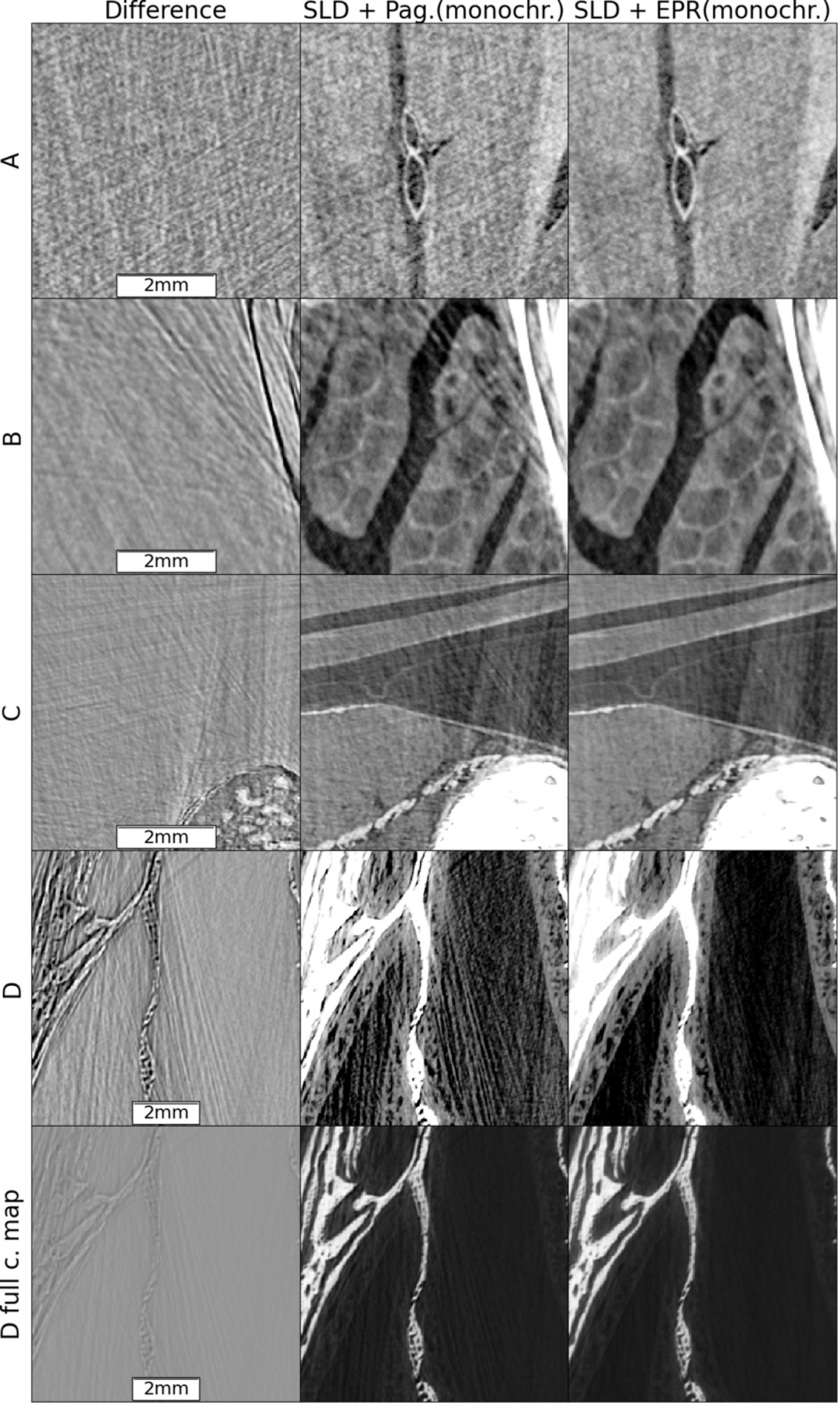
A detailed comparison between the EPR and Paganin *et al.* methods on zoomed regions from Fig. 2 ▸ marked A, B, C and D. Monochromatic case.

**Figure 4:**
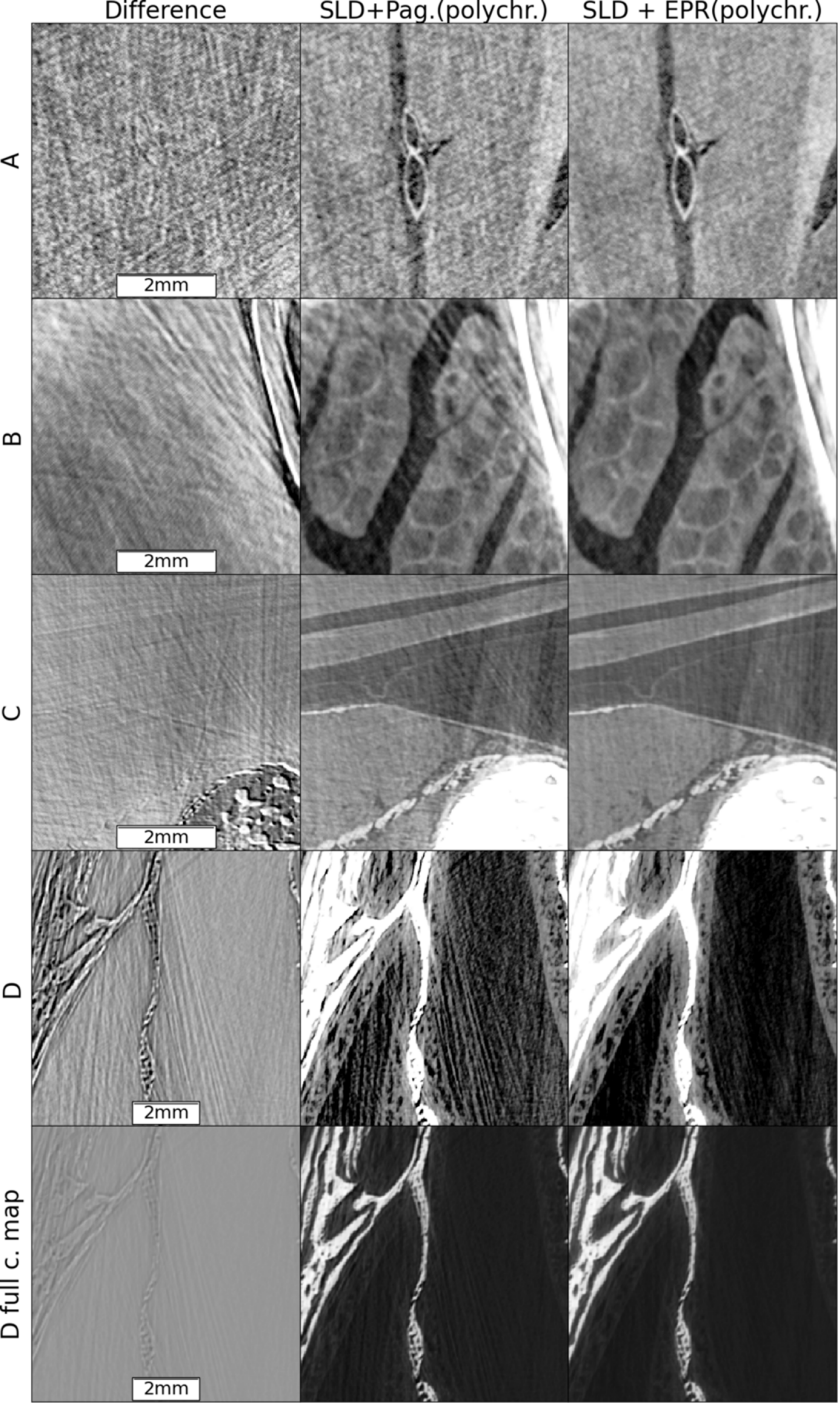
A detailed comparison between the EPR and Paganin *et al.* methods on zoomed regions from Fig. 2 ▸ marked A, B, C and D. Polychromatic case.

The spectra are calculated (Tafforeau *et al.*, 2023 ▸) given the experimental parameters for which a comprehensive view is provided in Table S1 of the supporting information. The vertical marks in Fig. 1 ▸ delimit the energy intervals, with each interval having the same integrated area, used for the spectral shape discretization in the polychromatic method discussed in the present work. Fig. 2 ▸ shows, for two slices of the sheep head, a comparison between the Paganin *et al.* algorithm (Paganin *et al.*, 2002 ▸) coupled with an unsharp mask filter (Sheppard *et al.*, 2004 ▸) applied to the radiographs, after the phase retrieval, to recover the fine structures (classical method for single distance PPC-µCT), and the result obtained by applying all the improved methods introduced by the present work. At the scales visible in Fig. 2 ▸, two major improvements can be observed: the complete correction of the bright halos surrounding the absorbing features and a reduction of the beam-hardening-like artifacts. These improvements are the effect of the SLD method. The improvement brought by the EPR method becomes visible when zooming to smaller length scales. Specifically, the EPR corrects the strong gradient effects which occur on small length scales. For a closer examination of EPR corrections, Figs. 3 ▸ and 4 ▸ provide zoomed views of the insets A (brain), B and C (soft tissues near the bones) and D (nasal mucosa). The gray scales used have a constant minimum to maximum width over a given row and are centered, for the four top lines, over the soft matter range. The comparison is done twice, first in Fig. 3 ▸ considering a monochromatic beam at the average 109 keV energy, and with a delta–beta ratio of 992, and then in Fig. 4 ▸ for the polychromatic case. The reason for detailing the two cases separately is that, otherwise, when taking the difference between the two methods without considering the same spectral shape, the absolute scales of the gray levels would differ due to beam hardening, albeit slight here, which is implicitly accounted for in the polychromatic treatment but not in the monochromatic one. The polychromatic version of the Paganin phase retrieval has been implemented replacing the EPR forward model, for a given spectral point, by equations (1) and (2), and keeping the rest of the EPR code unchanged.

The nasal mucosa is shown twice: first, on the fourth line, shrinking the gray scale window to capture the mucosa, then using the full range to show the bone details. Interestingly, the difference image is weakly correlated with the image soft regions features but strongly with the bone interfaces, especially in tangential directions. These regions typically experience strong gradient, and consequently our algorithm corrects most strongly in these regions. The long range of these artifacts is also noteworthy. Despite being up to 400 pixels from the bones, the errors removed from region A have a visibility comparable with that of the imaged features. Region C remains the most problematic one, as artifacts corresponding to the long absorption path through elongated bones are not fully corrected. The improvements, highlighted by the region D insets, illustrate the practical benefits of the EPR algorithm. Here a simple threshold-based segmentation method, saturating the bone regions and under-saturating the interstitial ones, if used on the EPR images, is enough to capture the mucosa whose profile would otherwise be lost.

**Figure 5:**
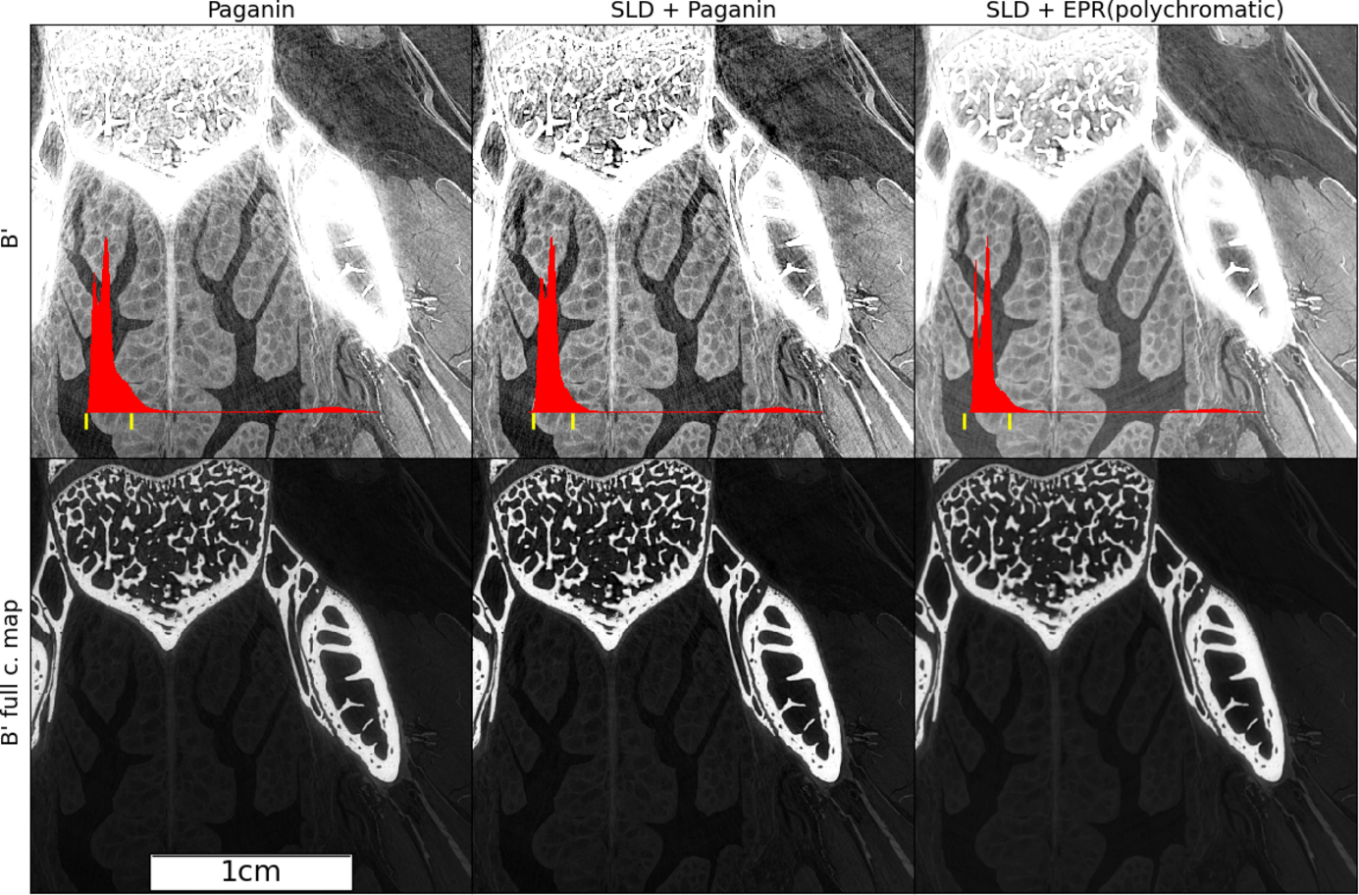
Incremental application of SLD and EPR: (left) reconstruction by the standard Paganin *et al.* algorithm, (center) the result by preprocessing the radiography with SLD, (right) the effect of both the SLD and EPR algorithms. In the first row the gray scale is adapted to the soft matter (the range is marked by yellow ticks in the red histogram); in the second it covers the whole range.

**Figure 6:**
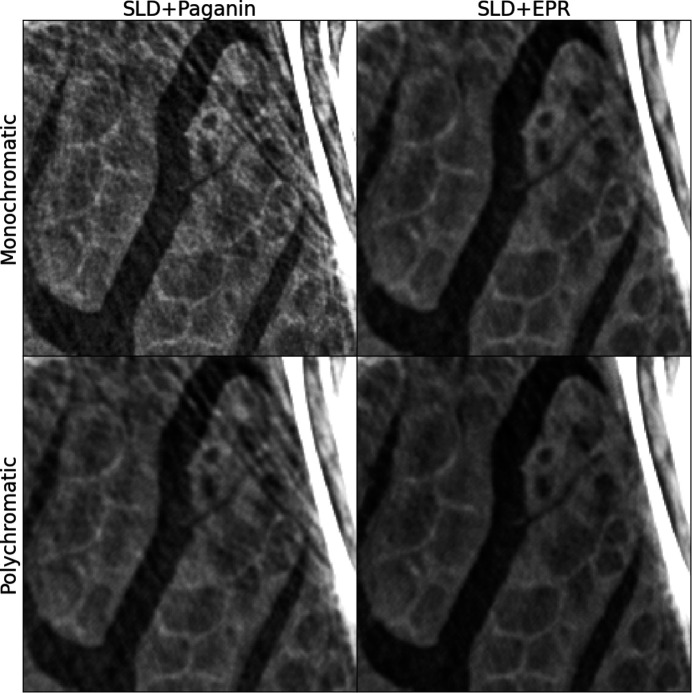
Effect of the polychomatic method for EPR and Paganin. First row: monochromatic approach considering only one photon energy in both the Paganin and EPR forward model. Second row: polychromatic approach considering the discretized spectra, with five photon energies (as in Fig. 1 ▸), in the forward models. The polychromatic version of the Paganin phase retrieval has been implemented replacing the EPR forward model, for each spectral point, by equations (1) and (2), and keeping all the rest of the EPR code unchanged.

To illustrate the joint effects of the two algorithms (SLD and EPR) we use an intermediate scale in Fig. 5 ▸ where we show a region *B*′ which is larger than and centered on region B. The figure illustrates the reconstruction by the standard Paganin *et al.* algorithm (left), the result obtained preprocessing the radiographs with SLD (center), and finally the effect of both SLD and EPR algorithms (right). While the SLD deconvolution effectively removes the bright halo surrounding the bones, the EPR method’s effect (removal of streak artifacts) remains effective and visible even far from the bones which are the source of the artifact. Notwithstanding the above, pointing toward the role of the strong gradient and the breaking of the linearization approximation, one might still wonder whether the observed improvement could be ascribed instead to the accounting of polychromaticity done in EPR. To answer this question we show in Fig. 6 ▸ a comparison of the Paganin algorithm with EPR for both a monochromatic and polychromatic beam spectral model. We can observe that the polychromatic version of the Paganin forward model still produces all the streak artifacts which are instead suppressed at a higher degree by a merely monochromatic EPR algorithm.

**Figure 7:**
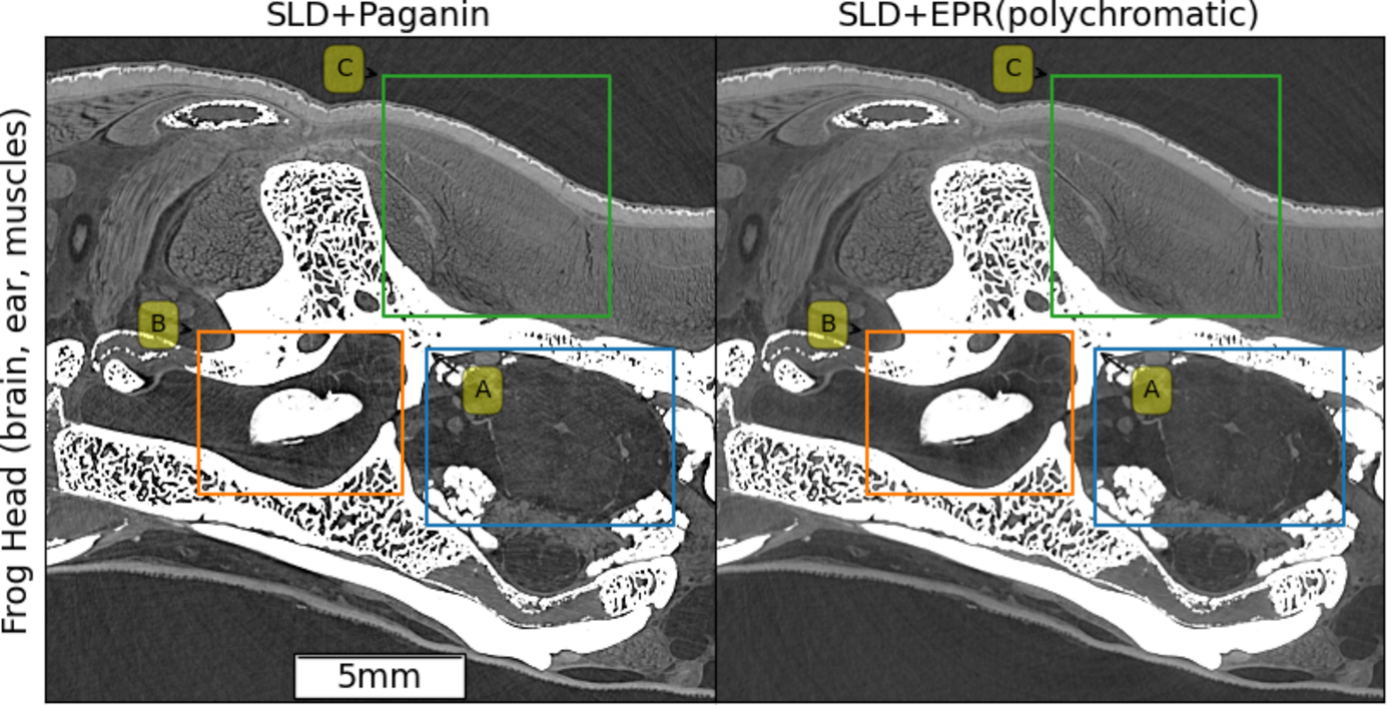
Frog head: 900 × 800 view spanning the brain (A), ear (B) and skull muscles (C) subregions. The image has been obtained with SLD + Paganin (left) and EPR (right) algorithms. The acquisition has been performed at BM18 with an average beam energy of 127 keV using the HiP-CT protocol, a voxel size of 23.27 µm and 20 m propagation distance.

**Figure 8:**
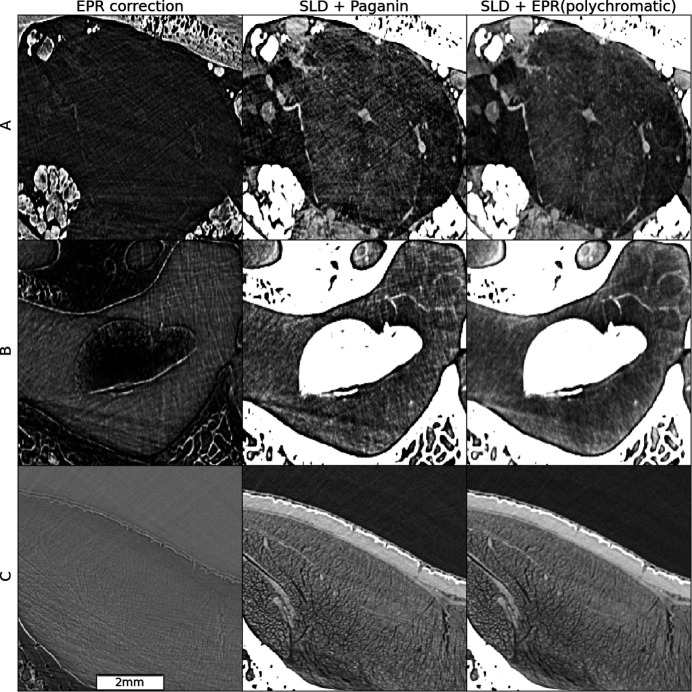
Zoomed views of the frog head subregions. Rows: regions A, B and C from Fig. 5 ▸. Columns: the difference between the two methods (left); SLD + Paganin *et al.* (center); SLD + EPR with five spectral points (right). The used gray scales have a constant minimum to maximum width over a given row, and are centered, for each image, over the soft matter range.

**Figure 9:**
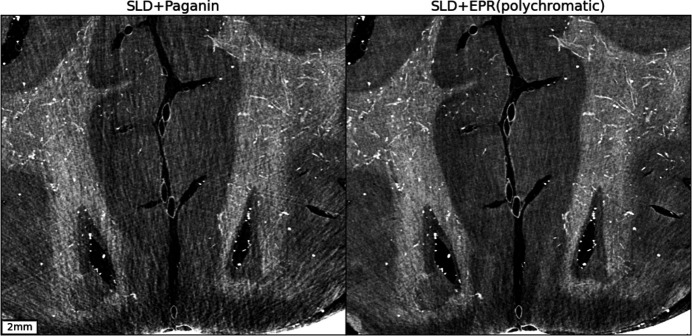
Sheep head HiP-CT scan at 112 keV with a 16.45 µm detector pixel size and 30 m propagation distance. The shown region corresponds to region A of Fig. 4 ▸. Reducing the pixel size (from 28 µm of Fig. 4 ▸), while keeping unchanged the propagation distance, enhances the phase effects but also the linearization artifacts which remains strongly reduced using the EPR algorithm.

Fig. 7 ▸ shows a slice subregion from the giant frog head. The acquisition was performed at BM18 with an average beam energy of 127 keV, a voxel size of 23.27 µm and a propagation distance of 20 m using the HiP-CT protocol. A detailed comparison between the classical Paganin *et al.* approach and EPR is shown in Fig. 8 ▸ for regions A (cerebral region midbrain), B (membraneous vestibular region of the inner ear (saccular otoconia) and C (dorsalis scapulae muscle). The brain is contained in a mainly convex region and, for this reason, its reconstructed image is not disturbed by those artifacts which are generated at the bones’ borders. They are tangential and do not cross the inner regions. In region A, the Paganin *et al.* reconstruction artifacts can be attributed to the bone trabecular regions, which, for certain directions, act as an array of refractive lenses. These artifacts are efficiently reduced by EPR. A similar artifact reduction is observed in the ear region. However, the bone tangential artifacts, now visible in this non-convex region, are only weakly reduced by EPR. Finally, in the muscles region (row C, Fig. 8 ▸), which has a higher signal contrast compared with other soft tissue areas, both Paganin *et al.* and EPR yield excellent results. For the frog head we have found that the effect of SLD is barely visible. This is due to the relatively small absorption of the skeleton in this sample. The absorption contrast observed in the radiographs between soft and bone regions is in fact always below 15%, as observed in the intensity modulations of the normalized radiographs. For the sheep head, instead, the contrast peaks at 70% in certain angles and regions. A practical aspect of the better image quality brought by EPR+SLD is that automatic segmentation is easier. This is visible already with a simple thresholding method as shown in Fig. 4 ▸, for region D. In the supporting information, the movies M1–M3, which explore the whole 3D volume of the sample, are provided as links to the ESRF data portal. The geometrical setup used for the sheep head tomography at a voxel size of 28 µm would have been challenging with the previous ESRF source. Specifically, with the ID19 beamline, which before the EBS-ESRF upgrade had a source horizontal FWHM size of 140 µm and a source–sample distance of 145 m, the confusion width at 30 m would have been 29 µm, comparable with the voxel size. In contrast, with the current BM18 beamline and the EBS beam’s horizontal size of 26 µm, the confusion width is only 4.5 µm which is negligible compared with the voxel size. This provides substantial flexibility to further enhance phase effects by further reducing the pixel size while maintaining a long propagation distance. Fig. 9 ▸ shows the sheep brain in region A of Fig. 4 ▸, obtained through a higher resolution scan with a 16.45 µm detector pixel size and 30 m propagation distance (see experimental parameters in Table S1 of the supporting information). Reducing the pixel size while keeping the propagation distance unchanged amplifies phase effects but also increases linearization artifacts associated with the Paganin *et al.* method, that remain effectively suppressed with the EPR algorithm.

**Figure 10:**
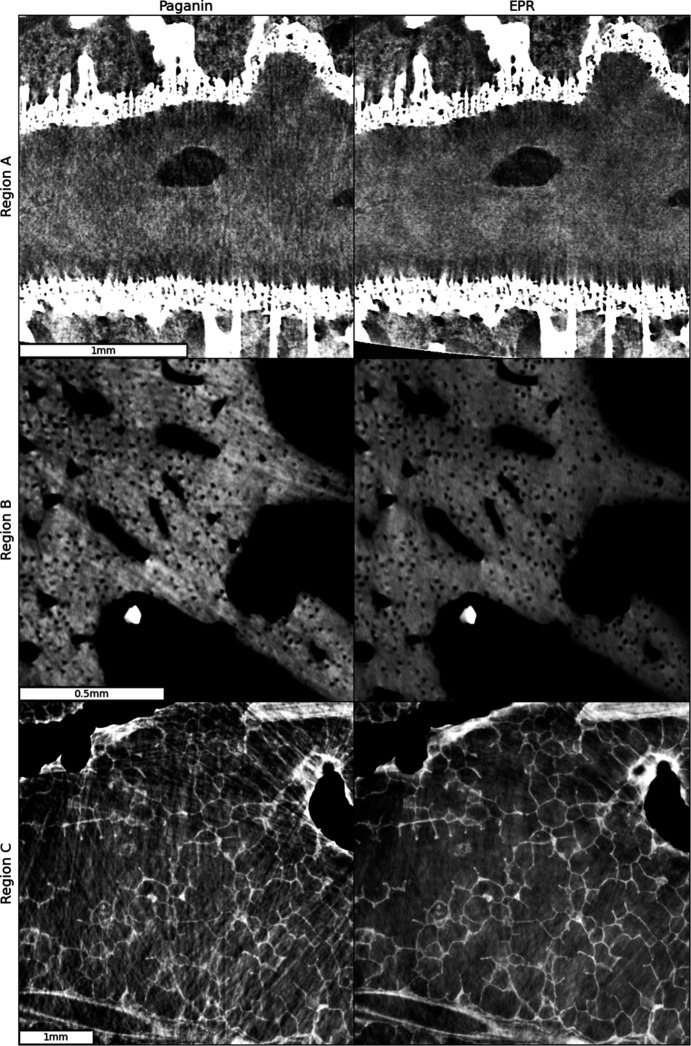
Comparison between the Paganin method and EPR. Region A: sheep head’s optic nerve region at 90 keV, 1.5 m propagation distance and 2.25 µm pixel size. Region B: rabbit bone at 103 keV, with 1.4 m propagation distance and 2.0 µm pixel size. Region C: an adult human lung sample region at 90 keV, 4 m propagation distance and 4.26 µm pixel size.

The phase-gradient-induced X-ray deflections, calculated using equation (3) during the application of equation (7) in an EPR run, peak at a maximum of two pixels for this scan. These shifts can be interpreted as a measure of the departure from the linearization approximation as discussed in Section 3.3. This maximum shift primarily occurs along paths near the long jaw bones, whereas in the brain region most artifacts arise from trabecular structures along much shorter paths. A closer inspection reveals that the associated shifts in this region are around 0.1 pixels or less. This suggests that the appearance of linearization artifacts does not require extreme phase enhancement conditions and may be observed even with more conventional experimental setups. Fig. 10 ▸ shows insets from three scans performed under more standard conditions: a zoomed-in view with a 90 keV polychromatic beam, a 1.5 m propagation distance and 2.2 µm pixel size of the sheep head’s optic nerve region; a rabbit bone at 103 keV with a 1.4 m propagation distance and 2.0 µm pixel size; and an adult human lung sample region at 90 keV average energy, a 4 m propagation distance and 4.26 µm pixel size (see experimental parameters in Table S1 of the supporting information). These parameters can be achieved on several high-energy micro-tomography beamlines at third-generation synchrotron sources. The insets demonstrate the EPR algorithm’s suppression of linearization artifacts originating in the sheep head from trabecular structures, in the rabbit bones from the bone inner cavities, and in the adult human lung from bubbles in the 4% formalin embedding medium, an unintended effect of the sample treatment. In all cases, we observe significant suppression of linearization artifacts. The complete slices from which the insets have been extracted are shown in Fig. S1.1 of the supporting information.

**Figure 11:**
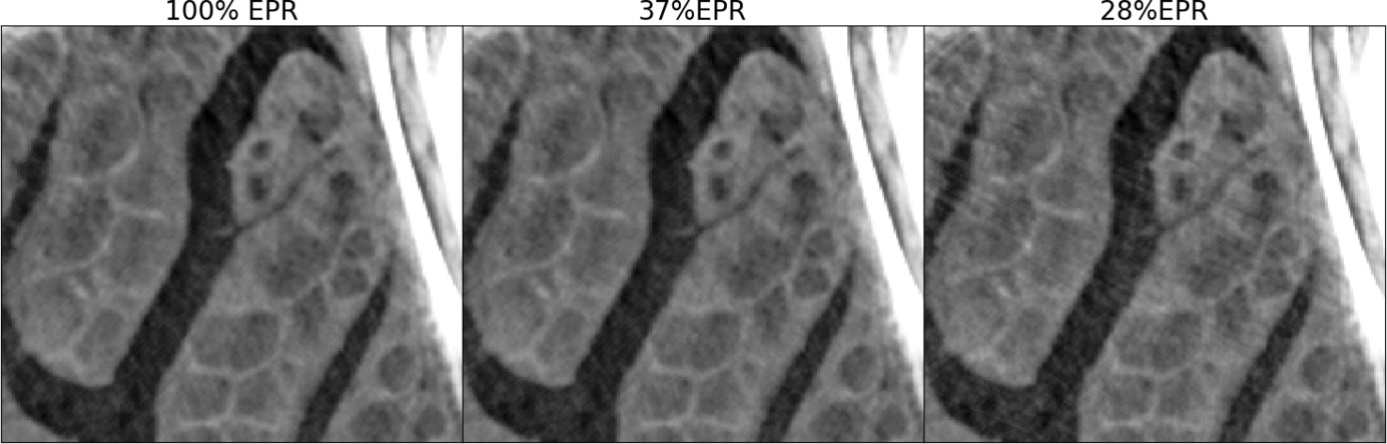
Hybrid scheme results, where only a fraction of each radiography is processed with EPR and the remaining part with Paganin, according to automatic detection of the critical regions. From left to right the results are shown for an overall average of 100%, 37% and 27% of the total projection volume processed with EPR. For an EPR coverage of 37% the difference with the 100% case is barely visible.

The artifacts have their origin only in particular regions of the radiographs, which are projections, for certain angles of certain strong gradient sample regions. Fig. 11 ▸ shows the application of this method, obtained by classifying with machine-learning the most-critical regions of the projections (Admans, 2024 ▸). These preliminary tests show that we can cut, by at least by a factor of three, the computing cost.

## 3. Material and methods

The widely used retrieval algorithm by Paganin *et al.* (Paganin *et al.*, 2002 ▸) relies on the linearization of the intensity evolution along the propagation axis. To illustrate the limitations of this approximation when strong phase effects occur, we briefly review its framework.

### 3.1. The Paganin *et al.* algorithm

For a point in the wavefront plane, the intensity at the detector, placed at *z* = *L*, is written, for a parallel beam, as 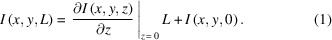
Here the propagated intensity is written, for a point in the wavefront plane, as the sum of the intensity at position (*x*, *y*, *z* = 0) and a term obtained by multiplying the propagation distance with the derivative of the intensity with respect to *z* at the same position. This derivative is obtained, in the following equation, from the transverse derivatives of the intensity and of the phase by applying the transport-of-intensity equation (TIE) (Teague, 1983 ▸),
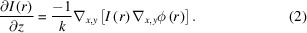
Eventually equation (1) takes an easily invertible diagonal form after Fourier transformation, assuming the local refracting index ratio δ(*r*)/β(*r*) remains constant throughout the sample (Paganin *et al.*, 2002 ▸). Then, considering an incoming plane wave of intensity *I*_0_, immediately after the sample, ϕ(*x*, *y*, 0) = (δ/β)log[*I*(*x*, *y*, 0)/*I*_0_]/2 and the right part of equation (2) becomes proportional, for *z* = 0, to the in-plane Laplacian of *I*(*x*, *y*, 0). For the cone beam case, a preliminary transformation to the parallel geometry case must be applied (Pogany *et al.*, 1997 ▸), after which the same formalism applies.

### 3.2. A preliminary observation

Notably, we describe the following observation that will be used to illustrate later the linearization failure: considering a subregion of the (*x*, *y*, *z* = 0) plane, with a hypothetical constant intensity *I* and phase ϕ, equation (1) propagates its wavefront to the same subregion at *z* = *L* with the same value of *I*. This is because, from equation (2), ∂*I*/∂*z* = 0 for that region.

### 3.3. Limitation for strong gradients

From a geometrical optics perspective, equation (2) can be interpreted as the effect of the angular deflection induced by the phase gradient. With respect to the impact point in the absence of the sample, this deflection induces a transverse shift of the photon impact point on the detector plane and, for constant δ/β, is equal to
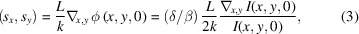
with 2π/*k* = λ being the radiation wavelength. The following simple conceptual experiment illustrates the limitation of the linearization approximation when the phase-gradient-induced shifts are larger than the size of the sample features. Assume that the intensity is *I* = 0 everywhere in the *z* = 0 plane except within a small circular spot. This can be conceptually realized with a beam going through a filter with pinhole whose thickness profile is zero at the pinhole center and increases rapidly in the border region. When the phase-gradient-induced shifts, at the spot border, are larger than the spot diameter, then photons passing by the region *I*(*x*, *y*, 0) ≠ 0 and with strong gradients, belonging to the spot border, will propagate to points (*x*′, *y*′, *L*), outside the geometrical projection of the spot, and therefore such that *I*(*x*′, *y*′, 0) = 0, but because of the deflected photons themselves it must be *I*(*x*′, *y*′, *L*) ≠ 0. These two latter conditions mutually exclude each other in the frame of equation (1), as in our preliminary observation.

### 3.4. The EPR algorithm

To overcome this issue, we first rewrite the TIE in a form which will inspire us in the development of our algorithm. We rewrite equation (2) as the continuity equation 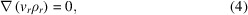
which expresses the conservation law for a gas of particles (the intensity loss due to absorption in air acts as a simple prefactor in paraxial approximation). In our case, the density is given by ρ_*r*_ = *I*(*r*) and the local average speed by the three components (∂_*x*_ϕ, ∂_*y*_ϕ, ∂_*z*_ϕ ≃ 2π/λ), where the third component is given in paraxial approximation. The equivalence between equations (2) and (4) already suggests the idea for a forward model by which we can replace equation (2) even in the non-linear case: it would be a kinematic diffusion of particles. Alternatively, we derive an equivalent formulation by writing the Huygens–Fresnel integral for the field at the detector position (*x*′, *y*′) in eikonal approximation. The integral is first written in its full form as
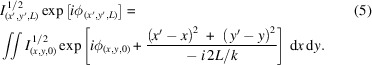
We approximate the result by summing only those eikonal paths defined by *s*_α_ = α′ − α, for α ∈ {*x*, *y*}, with the shifts *s*_α_, given by equation (3), for which the first derivatives in *x*, *y* of the optical path phase, which appears in the exponent, are zero. Contributions from the regions where the phase is rapidly varying are neglected. The non-essential longitudinal phase *kL* has been dropped from the definition of detector phase 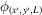, which does not play any role in the algorithm when the measured intensity is matched. Each eikonal path contributes then with a phase given by its optical path and with an amplitude depending on the second derivatives of the phase. These latter determine how narrow is the region where the phase is slowly varying. The approximation expresses the amplitude 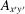 = 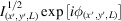, at detector position *x*′, *y*′, by the following equation,
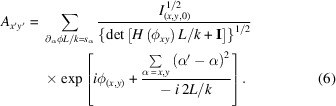
Here, the summation is performed, for a given detector point, over all the points satisfying equation (3), the eikonal condition. *H* represents the Hessian matrix, formed by the second derivatives of the phase. Finally, we obtain the forward model, which extends equation (1) to the non-linear regime, by obtaining the intensity at the detector as the modulus squared of the right member of the above equation, and discarding, in the near-field approximation, the interference between contributions of different paths,
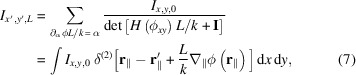
where we have collected the transverse coordinates into the vectors **r**_∥_ = (*x*, *y*), **r**′_∥_ = (*x*′, *y*′), and δ^(2)^ is the two-dimensional Dirac delta distribution. The algorithmic implementation of the forward model is dictated by the right member of the equation. The photons passing through each surface element d*x*d*y*, of the *z* = 0 wavefront, are collected at the pixel position (*x* + *s*_*x*_, *y* + *s*_*y*_). The justification for discarding the interference terms is based on the short longitudinal coherence length of the pink beam. In a near-field approximation, *d*^2^/*L* is larger than the typical wavelength when *d* is the distance between two points which are separated by more than one pixel width. Therefore, when we consider the shifts due to strong phase gradients, the interference terms, resulting from overlapping wavefront portions, likely originate from paths whose length differences are larger than the wavelength, and thus dis­appear. For the weak gradient regimes, where the Paganin *et al.* approach is still valid, two or more eikonal paths cannot interfere because this would require shifts larger than one pixel. The beam spectral width is therefore not a concern in this case.

The forward model is built on GPU and gives the simulated intensity at the detector as a functional of an effective absorption distribution. The code can be used for either polychromatic or monochromatic beams. The EPR algorithm incorporates a discretization of the beam’s spectral distribution. At each discrete energy, the refractive index is calculated from the Chantler tables (Klementiev *et al.*, 2023 ▸; Chantler *et al.*, 2023 ▸), considering the stechiometry. In monochromatic mode, and with a uniform unit illumination of the sample, the transmitted intensity that exits the sample is a function of the effective absorption *a*(*x*, *y*) that we take as the free variable of the forward model, which now takes as input *I*_(*x*,*y*,*z*=0)_ = 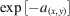, while the effective phase, used in equation (7), is deduced as ϕ(*x*, *y*) = − (δ_av_/β_av_)*a*_(*x*, *y*)_/2. Here the symbol ‘av’ indicates that the considered optical constants are taken at the effective average beam energy, averaged using the effective spectral shape as weight. These constants are calculated for the material of interest that produces the largest gradients leading to these artifacts, and then we subtract the refractive index of the embedding material. When our aim is correcting the streak artifacts originating at the bone interfaces we consider, in the calculation of the ratio, the bones’ refractive index after subtraction by the alcohol index, the latter being the embedding media. For the lung sample, instead, we have considered the interface between 4% formalin and vapor, neglecting the density of the latter.

### 3.5. EPR with polychromaticity

When polychromaticity is considered, the whole spectral range is split into a user-chosen number of subranges, with the *i*th subrange being characterized by its spectral weight *f*_*i*_. The free variable of the forward model is still *a*_(*x*, *y*)_. The fractional intensity *I*_*i*_ of a given spectral range *i*, at the *z* = 0 plane, is derived from the effective beam spectral shape and materials optical constants,
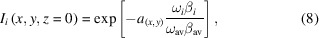
with ω_*i*_ being the subrange weighted average of the photon energy and β_*i*_ the refractive index imaginary part at ω_*i*_. The phase for subrange *i* is
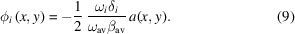
The variable *a*(*x*, *y*), a function of the wavefront position, is found by the Conjugate Gradient (CG) algorithm, minimizing the *L*_2_ norm of the difference between the data and the forward model. The comparison reconstructions, carried out using the Paganin *et al.* method, are performed with a delta–beta ratio equal to δ_av_/β_av_.

The implementation of the forward model is based on splitting the wavefront into pixels; then, based on the phase gradient, the corners of each pixel are ray-traced onto the detector, over which their intensity is distributed by pixel-splitting. This forward model is robust against strong phase gradients. In the cases treated in this work, the retrieved phase differences between two adjacent pixels can reach values of the order of *N*2π with *N* ≃ 3 in the regions with the strongest gradient. Because of the magnitude of these phase steps, faster forward models based on the fast Fourier transform (FFT) (Mas *et al.*, 2003 ▸) would require heavy oversampling, which would likely cancel the benefits of the FFT—at least for the present cases. Note that in the Paganin method, which also uses FFT, phase wrapping does not occur because the TIE propagates a real-valued function.

As the forward model is not a simple linear operator, convergence with the CG method is not guaranteed. Moreover, the problem suffers from a high condition number: high spatial frequencies converge more rapidly than low frequencies, leading to zigzag instabilities around sharp edges. To address this, we employ a Hierarchical Preconditioning scheme. We proceed through *N* spatial scales, starting from a coarse, rescaled problem with pixel size 2^*N*^ and refining down to the fully resolved scale. At each level, we oversample the solution from the previous (coarser) scale and use it as the initial guess for the next. For the 2^*N*^ scale the number *N* is taken large enough so that the phase effects, at this scale, are negligible and the solution trivial. For all the examples shown in this work, *N* = 7 scale levels were applied with an *a priori* chosen number *N*_CG_ ≃ 30 of CG iterations applied for each level.

The above techniques constitute the core of the EPR algorithm, but also contribute to its numerical cost. Unfortunately, in the field of phase retrieval, iterative algorithms are typically necessary due to the inherently ill-posed nature of retrieving phase information from intensity measurements alone. Analytical methods, enabling direct inversion without iteration, represent notable exceptions under specific favorable conditions.

A well known example is Paganin’s method, which exploits the linearization of the wavefront differences between the sample and detector planes. This method leverages the TIE, employing a notable mathematical simplification: the spatial derivative (in-plane derivative) of the logarithm of the measured intensity results in a denominator containing the intensity itself. Crucially, this denominator cancels out the intensity factors, ensuring a linear expression in intensity and thus simplifying the reconstruction significantly. Without this particular mathematical ‘trick’, possible in the constant δ/β approximation, the intensity would appear multiple times, making the relationship inherently nonlinear and complicating direct inversion.

Another significant analytical approach is the multi-distance phase retrieval method introduced by Peter Cloetens and colleagues (Cloetens *et al.*, 1996 ▸). This technique assumes conditions of weak absorption, allowing linearization of the phase–intensity relationship in Fourier space. Under these assumptions, the resulting expressions remain first-order linear in the intensity, enabling a straightforward analytical reconstruction without iteration.

### 3.6. The SLD algorithm

The presence of strong gradients in both the phase and absorption creates another kind of artifact attributed to the secondary light propagation inside the scintillator and its subsequent scattering. The scintillator, an integral part of the experimental setup, is responsible for converting X-rays into visible light. However, a fraction of this light, instead of traveling straight to the imaging optics, remains trapped due to total internal reflections inside the scintillator plate. It then travels until it is either absorbed or scattered, leading to a diffused background around the bright regions of the radiographs, thus altering the values of neighboring regions. In order to deconvolve this signal, we postulate that the point spread function *p*_*i*_(*j*), *i.e.* the signal recorded by the detection setup at a pixel *j*, for a photon which scintillates at the position corresponding to pixel *i*, located at a distance *r*_*ij*_ from pixel *j*, is
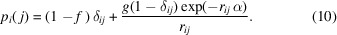
The case of a perfect scintillator, without diffused light, would correspond to *f*, *g* = 0. In this case, one recovers the ideal spread function *p*_*i*_*j* = δ_*ij*_. A real, non-ideal, scintillator is characterized by a non-zero *f*. This parameter is the fraction of the total collected light which is found in the tail. The parameter *g* is set by the condition that the sum of *p*_*i*_(*j*) on all the pixels is normalized to 1. The functional form of the tail contains the 1/*r* decrease of light intensity in 2D geometry, plus the damping exp(−*r*_*ij*_α) due to the light escaping from the scintillator surfaces or being absorbed. The free parameters are *f* and α. To determine these parameters, with the same experimental setup that is used for the tomography experiment, we form a bright small spot on the scintillator by collimating the X-ray beam with slits. Then the spot halo is fitted with the model. The obtained function is then used to deconvolve the raw data. Finally, for all the reconstructions shown in this work, an unsharp filter (Sheppard *et al.*, 2004 ▸) has been applied, for every retrieved absorption radiography, following the HiP-CT protocol (Walsh *et al.*, 2021 ▸), with a sigma of 1.2 pixels for the blurring Gaussian kernel and a multiplicative coefficient equal to 4. The EPR retrievals have been performed considering a beam spectral splitting consisting of five contiguous spectral regions, covering the whole energy range, each having the same integrated area.

## 4. Conclusion

Our contribution to image quality improvement is twofold and concerns both low and high frequency artifacts. Concerning the low frequency artifacts, the improvement is based on a simple ansatz for the scintillator response, similar to the approach presented by Vo *et al.* (2019 ▸). The strong improvement in the image quality may justify, in the future, more in-depth characterization of each scintillator properties, but already our simple implementation has proven to be very effective.

Concerning the high frequency artifacts, here lies the most original part of our contribution. We deeply revise one of the most used algorithms in synchrotrons and propose an alternative formulation which copes with large beam deflections. We show drastic suppression of those artifacts which appear as lines originating from strong absorption gradient regions. We stress that our method is based on an improved physical modeling of the beam propagation and simply improves the retrieved signal without artificially post-filtering out any part of it.

However, it is worth noting that some residual artifact remains. For instance, in Fig. 4 ▸, rows A and B, the EPR images are still crossed by arrays of residual faint lines, although their visibility has much decreased compared with the classical Paganin *et al.* images. Moreover, residual artifacts remain strong in regions characterized by long absorption paths running tangentially to large bones’ thicknesses (Fig. 4 ▸, row C). This trend is confirmed in the frog sample where the artifacts originating from trabecular structures are strongly suppressed, while the bones’ tangential artifacts are only weakly reduced. Our hypothesis is that these residual artifacts arise due to the lack of representability of those gradients which occur on slope widths smaller than one pixel. These gradients are underestimated by the discretized numerical procedure which sees them occurring over one pixel. However, despite the residual artifacts, the improvements in image quality are substantial and demonstrate that we are truly leveraging on the inherent physics underlying the observed phenomena. The comparison with the Paganin *et al.* algorithm, both monochromatic and polychromatic, highlights the considerable improvement that EPR can bring to the state-of-the-art PPC-µCT imaging at modern synchrotrons. Beside the esthetic aspects, our algorithm recovers features, as shown in region D of Fig. 4 ▸, which were lost under the now corrected artifacts. Concerning the residual artifacts, we can base our future strategy, for further improvements, on the logic about gradient representability. Suppose we are interested in a given resolution goal, then the acquisition could be performed at higher resolution. Reducing the pixel size decreases the counts per pixel, with the drawback of increasing the statistical errors. The proper statistics can then be retrieved by transforming the reconstructed image to the lower resolution goal. In fact, it is the signal originating from the soft matter, having the lowest contrast, which is the most affected by the noise. On the other hand, this signal is weak and thus does not create non-linearities in the retrieval procedure. The errors will therefore cancel, with random signs, when we revert to lower resolution after the phase retrieval, thus recovering the expected statistics for the goal resolution. The interesting point is that the problematic signal originates from strong gradient regions and these will be better represented, using higher resolution, during the phase retrieval, while their signal is strong and thus more robust to noise. We have shown that the linearization artifacts are visible also at conditions corresponding to more standard high-energy micro-tomography beamlines, with propagation distances of a few meters. Future work aims to extend these studies to lower energy scans, potentially reaching energies as low as approximately 30 keV. The visibility of the linearization artifacts does not depend only on the magnitude of the X-ray deflection, and can be visible also for sub-pixel shifts. If we consider a perfectly circular structure then a linearization error will occur all around the structure border, where the gradient is stronger, but in the reconstruced image this error will be uniformly back-projected over 2π and thus it will not be detected as streak lines but as a non-ideal step-like shape of the edges. If now we consider two circular structures which are distant from each other, the same argument can be applied separately to the two structures as long as we consider that the inverse radon transformation is a linear operator, and one could expect, based on this simplified argument, that the reconstruction would give just the sum of the two structures each one without visible streaks. However, it occurs that when, during the sample rotation, the same X-ray path is tangent to both structures, the wavefront phase gradient, for that ray, doubles, but the second-order effects on the observed signal are multiplied by four, instead of two. This breaks the linear­ization picture. For this angle a streak will appear in the reconstructed image. All this illustrates how errors, without treating the non-linear phase effects, are always present but their visibility depends on the geometrical details of the sample. Regarding the choice of delta–beta parameters, these are computed for the interfaces exhibiting the strongest phase gradients. Consequently, for bone interfaces the delta–beta ratios used are lower than those for soft-matter regions. This poses no issue for high-energy beams, since Compton scattering reduces the effective differences between delta–beta ratios. If the algorithm is applied at lower energies, a pre­liminary reconstruction is performed using EPR with a delta–beta ratio tuned to the bones. This yields a volume in which the soft-matter regions are processed with a reduced-delta–beta Paganin filter and thus display enhanced high-frequency content due to a sharper Paganin denominator in the frequency domain. Optionally, this enhancement can be corrected by segmenting the soft-matter regions and applying a dedicated correction filter to them. Concerning computing resources, both datasets (for the sheep head and the frog) had a size order of half a terabyte each. When fully processed by the EPR algorithm the computing time was, for one dataset, of the order of one day and a half, using 12 NVIDIA A40 GPUs. In comparison, the actual maximum throughput of the beamline is of the order of one similar sample every 4 h. However, not all the measured samples require the EPR algorithm. Moreover, when present, the artifacts have their origin only in particular regions of the radiographs, which are projections, for certain angles of certain strong gradient sample regions. In an ongoing development, the EPR algorithm will be used only in such regions; the Paganin *et al.*, with the SLD, will continue to be used for all the non-critical ones which are the majority of the data, thus reducing the computing time and making the EPR algorithm usable on a routine basis with moderate resources. Moreover, given the broad applicability of our algorithm, further work is underway to replace EPR with a trained U-Net network. This substitution aims to further reduce computational costs, enabling the new algorithm to be used routinely across all scans.

## Supplementary Material

Supporting information. DOI: 10.1107/S1600577525005223/tol5010sup1.pdf

## Data Availability

The code sources to reproduce the shown images and a data subset can be retrieved from the gitlab portal (Mirone, 2023 ▸), under MIT licence, and from the ESRF data portal (Mirone *et al.*, 2023 ▸), respectively.
