# Supplementary material for: Eikonal Phase Retrieval: unleashing the potential of fourth-generation sources for enhanced propagation-based tomography on biological samples
Source: J Synchrotron Radiat. 2025 Jul 17;32(Pt 5):1291–301. doi: 10.1107/S1600577525005223 (PMC12416417; doi:10.1107/S1600577525005223)
Supplement: Supplementary file 1 [file s-32-01291-sup1.pdf]

JOURNAL OF SYNCHROTRON RADIATION

Volume 32 (2025)

Supporting information for article:

Eikonal Phase Retrieval: unleashing the potential of fourth-generation sources for enhanced propagation-based tomography on biological samples

Alessandro Mirone, Joseph Brunet, Theresa Urban, Hector Dejea, Leandre Admans, Renaud Boistel, Morgane Sowinski, Pierre Paleo, Henry Payno, Stijn E. Verleden, Camille Berruyer, Elodie Boller, Claire L. Walsh, Peter D. Lee and Paul Tafforeau

1. Supporting Informations

1.1. Movie M1.

Movie M1 referes to File movie_X3_combined_HD.avi

Please find it at https://data.esrf.fr/doi/10.15151/ESRF-DC-1415285771 The video shows a 3D exploration, in parallel for three algorithms, of the sheep head. From left to right visible are : 1) Paganin, 2) SLD + Paganin, 3) SLD +EPR. The effects of SLD are always visible, around the absorbing features. The effects of EPR are visible mainly toward the end of the movie, in the nasal mucosa region, and in the brain region in the cerebellum.

1.2. Movie M2.

Movie M2 referes to File movie_X3_combined_HD.mp4

Please find it at https://data.esrf.fr/doi/10.15151/ESRF-DC-1415285771 Video M2 is the compressed version (mp4 format) of video M1.

1.3. Movie M3.

Movie M3 refers to Sheep-head_MRI-HiP-CT_V2_100s.mp4

Please find it at https://data.esrf.fr/doi/10.15151/ESRF-DC-1415285771

The video starts with a comparison, for the sheep head, between left) MRI and right) HiP-CT with SLD and EPR. Then the video first explores volume slice-wise manner and, at about 2/3 of the duration, it starts showing segmented 3d organs.

Table S1: Overview of the experimental parameters used in this work

Sample	Sheep head	Sheep head	Leptodactylus pentadactylus IRSN-391F	Sheep head ROI	Bubbly lung A186 ROI	Rabbit Bones
Optic	Lafip 2 (x0.125)	Lafip 2 (x0.25)	dzoom Hasselblad (x0.24)	Tandem 2x Otus Nikkor	Tandem 1x Otus / Otus	Tandem 2x Otus Nikkor
Voxel size (µm)	27.73	16.54	23.27	2.203	4.26	2.2
Date / Proposal	md1290 20/11/2022	md1290 21/06/2024	ls3105 10/06/2022	blc15662 10/09/2024	md1389 11/07/2024	commissioning
Average detected energy (keV)	108	112	131	96	92	102
Filters (mm)	Mo 0.44	Sapphire 10, Ag 0.3	Mo 1.3	Sapphire 1, Mo 0.44	Sapphire 5, Ag 0.1	Sapphire 1, Ag 0.5
Water equivalent surface dose rate on ROI	26.8 Gy/s	14.7 Gy/s	13.1 Gy/s	13.4 Gy/s	8.9 Gy/s	9.1 Gy/s
Absorbed dose by the bulk sample	16.9 kGy	18 kGy	15.3 kGy	26.4 kGy	23.5 kGy	24 kGy
Propagation distance (m)	30	29.4	20	1.5	4	1.4
Sensor	iris15	iris15	sCMOS PCO edge 4.2	iris15	iris15	iris15
ROI (HxV)	3104*256	5056*600	2048*400	5056*2960	5056*1400	5056*1400
Scintillator	LuAG:Ce 2mm	LuAG:Ce 2mm with reflective layer	LuAG:Ce 2mm	LuAG:Ce 50µm with reflective layer	LuAG:Ce 100µm with reflective layer	LuAG:Ce 50µm with reflective layer
X-ray source	BM18 lateral beam (1.08T)	BM18 lateral beam (1.08T)	BM18 lateral beam (1.08T)	BM18 lateral beam (1.08T)	BM18 lateral beam (1.08T)	BM18 central beam (1.56T)
Machine filling mode	200 mA	200 mA	200 mA	200 mA	200 mA	75 mA
Projection number	8000	15000	6000	15000	9000	8000
Scan geometry	HiP-CT in 70% ethanol using 140mm diameter jar immersed in 150mm tube, 360°, half-acquisition 1100 pixels, vertical series 5 mm	HiP-CT in 70% ethanol using 140mm diameter jar immersed in 150mm tube, 360°, half-acquisition 2000 pixels, helical scan with 5 mm vertical displacement	HiP-CT in 70% ethanol using 100mm diameter jar without immersion, 360°, half-acquisition 900 pixels, vertical series 7 mm	HiP-CT in 70% ethanol using 140mm diameter jar in 150mm tube, 360°, half-acquisition 2000 pixels, helical scan with 3 mm vertical displacement	HiP-CT in 4% formalin using 200mm diameter jar immersed in water in a 215mm tube, 360°, half-acquisition 500 pixels, helical scan with 2.9 mm vertical displacement	Classical propagation phase-contrast scan on 360 degrees
Subframe time (s)	0.006	0.008	0.02	0.035	0.04	0.05
Exposure time (s)	0.03	0.008	0.08	0.035	0.04	0.05
Accumulation level	5	1	4	1	1	1
Time per scan (min)	4.9	2.4	9.3	9.6	6.6	7.3
Number of scans	44	43	29	6	6	1
Total time (h)	3.6	1.7	4.5	1.1	0.7	0.1
Classical reconstruction protocol	HiP-CT normalisation by reference jar, single distance phase retrieval with filtered back-projection, vertical concatenation, 16 bits conversion, ring artefacts correction, jp2 conversion	HiP-CT normalisation by reference jar, single distance phase retrieval with filtered back-projection, helical reconstruction, 16 bits conversion, jp2 conversion	HiP-CT normalisation by reference jar, single distance phase retrieval with filtered back-projection, vertical concatenation, 16 bits conversion, ring artefacts correction, jp2 conversion	HiP-CT normalisation by reference jar, single distance phase retrieval with filtered back-projection, helical reconstruction, 16 bits conversion, jp2 conversion	HiP-CT normalisation by reference jar, single distance phase retrieval with filtered back-projection, helical reconstruction, 16 bits conversion, jp2 conversion	HiP-CT normalisation by reference jar, single distance phase retrieval with filtered back-projection, helical reconstruction, 16 bits conversion, jp2 conversion

1.4. *Experimental parameters TEST.*

1.5. *Scans at low propagation distance*

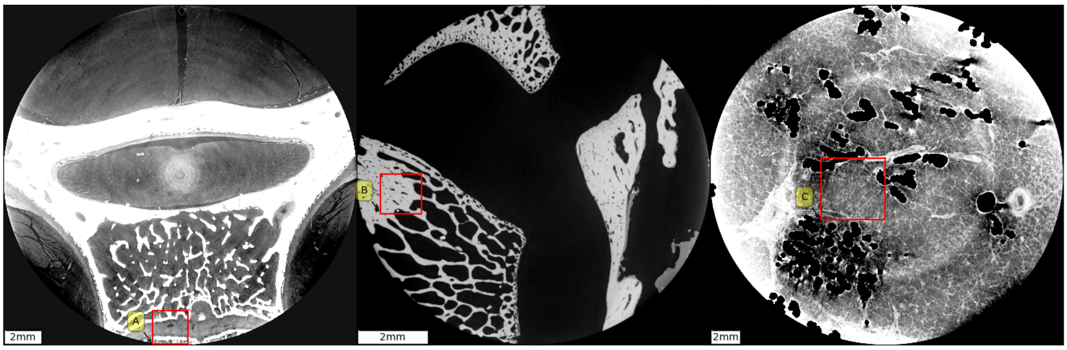

Fig. S1.1. Left: a zoomed-in view at 90 keV polychromatic beam, with a 1.5 m propagation distance and 2.2μm pixel size of the sheep head's optic nerve region. Center: rabbit bones at 103 KeV with 1.4m propagation and 2.0 andμm pixel size. Right: a adult human lung sample region at 90 keV average energy, 4 m propagation distance and 4.26μm pixel size. The red contoured insets are shown in Figure 10 comparing the Paganin et al. phase retrieval with EPR

1.6. *Instructions for testing the algorithm on a small synthetic dataset*

Besides the sheep-head dataset, the reader can test the algorithm on a small synthetic dataset by following the steps below. The dataset is generated using the script `demo_build_shape.py`, which is included in the `aspect_phase` repository. This synthetic dataset (a few MB) serves as a lightweight alternative to the 20 GB dataset available on the ESRF data portal.

1.6.1. *Prerequisites* Ensure the following:

- A Linux machine with an NVIDIA GPU
- CUDA toolkit (`nvcc`) installed or loadable (e.g. via `module load cuda/11.8`)
- Python 3.8+ and a C/C++ compiler

1.6.2. *Set up the environment*

```
python3 -m venv ~/epr_testenv
source ~/epr_testenv/bin/activate
pip install wheel h5py scipy pyfftw matplotlib filelock pycuda pyopencl pybind11 pyunitsystem
pip install git+https://github.com/lebedov/scikit-cuda.git
pip install git+https://gitlab.esrf.fr/tomotools/nabu@v2024.1.1
git clone https://gitlab.esrf.fr/mirone/aspect_phase
cd aspect_phase
git checkout release_1
pip install .
```

1.6.3. Generate the dataset

```
source ~/epr_testenv/bin/activate
cd aspect_phase/demo_with_synthetic_dataset
python demo_build_shape.py
```

The script also accepts the optional flag `--weak_features` to simulate a sample with weak absorption. This reduces the contrast difference between Paganin and EPR retrieval.

1.6.4. Run EPR phase retrieval

```
./AspectApply.nrs | grep ERROR
```

Each line printed corresponds to a projection and lists the residual errors for each hierarchical scale. The sequence is pre-tuned (via `sequence.json`) to achieve convergence. If errors appear anomalous, rerunning the retrieval is usually sufficient.

1.6.5. Reconstruct

```
# Using EPR-retrieved data
nabu aspect_nabu.conf

# Using Paganin-retrieved data
nabu paganin_nabu.conf
```

subsubsection Visual comparison

```
imagej *.tiff
```

You can compare the two reconstructions slice by slice. With the default parameters —which correspond to relatively strong phase-contrast gradients—the effect of X-ray deflec-

tion is clearly visible. The single-distance Paganin formula corrects the lensing (curvature) term but does not compensate the prism (ray-deflection) term. When the X-ray beamlets are bent toward the more transparent regions, and this phenomena is not compensated by an adequate prims term, the shoulders of the intensity profile loose amplitude, producing an effective smoothing.

Running the example with the `--weak_features` flag, instead, reduces both absorption and phase shift: the deflection term becomes negligible and the Paganin formula recovers the correct profile shape. This behaviour is a clear manifestation of a non-linear effect: if the imaging process were strictly linear in absorption/phase, one would observe only a uniform rescaling and shape invariance.

The same mechanism explains the appearance of tangential streaks. When, during sample rotation, the beam becomes tangent to two interfaces whose gradients point in the same direction (both on the left or both on the right), the gradients add, doubling their strength and quadruplicating the second-order non-linear term; this produces a bright streak at that specific angle. Conversely, when the tangency involves two opposite gradients, the non-linear contribution cancels, resulting in a dark streak at that angle.

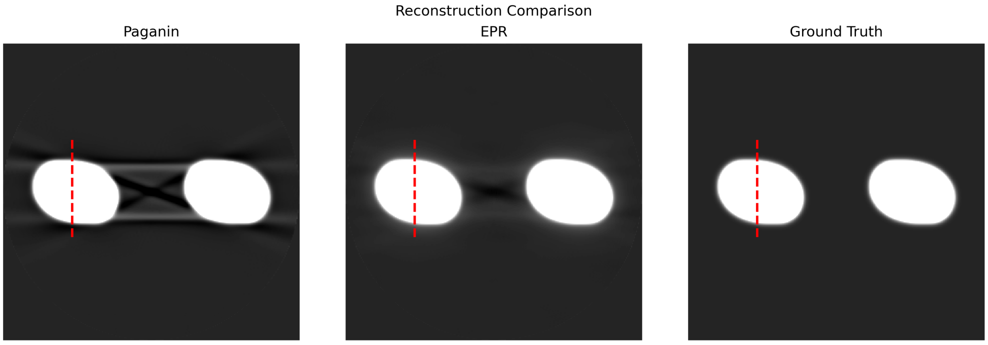

Fig. S1.2. Central slice of the synthetic dataset reconstructed with the Paganin filter (left), the proposed EPR method (centre), and the ground-truth phantom (right). A red dashed line indicates the column where the 1-D profile has been extracted. Display range is fixed between −0.02 and 0.12.

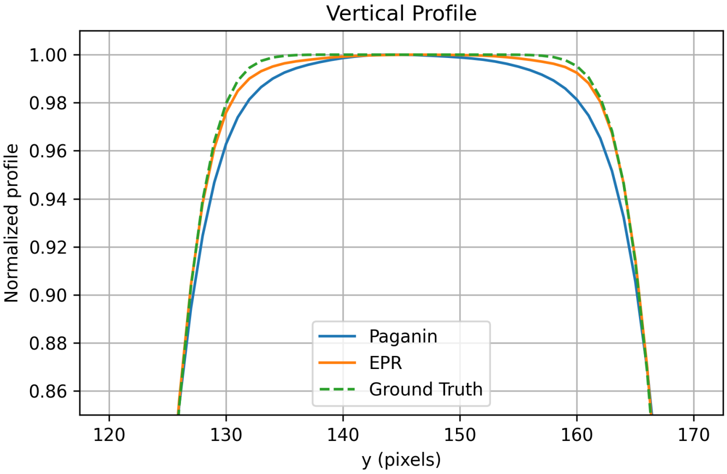

Fig. S1.3. Normalised vertical profile (50 px window centred on the maximum of the summed curves). The EPR retrieval preserves edge contrast, whereas, with the high phase gradient that are simulated in this test, the Paganin profile appears slightly smoothed.
